# The description of friction of silicon MEMS with surface roughness: virtues and limitations of a stochastic Prandtl–Tomlinson model and the simulation of vibration-induced friction reduction

**DOI:** 10.3762/bjnano.1.20

**Published:** 2010-12-22

**Authors:** W Merlijn van Spengen, Viviane Turq, Joost W M Frenken

**Affiliations:** 1 TU Delft, 3mE-PME, Mekelweg 2, 2628CD Delft, The Netherlands; 2 also with Falco Systems, Gelderlandplein 75L, 1082LV, Amsterdam, The Netherlands; 3 Université de Toulouse, UPS, INP, Institut Carnot Cirimat, 118, route de Narbonne, F-31062 Toulouse cedex 9, France; 4 Leiden University, LION, Niels Bohrweg 2, 2333CA, Leiden, The Netherlands

**Keywords:** MEMS, microscale friction reduction, normal force modulation, stochastic Prandtl–Tomlinson model, surface roughness

## Abstract

We have replaced the periodic Prandtl–Tomlinson model with an atomic-scale friction model with a random roughness term describing the surface roughness of micro-electromechanical systems (MEMS) devices with sliding surfaces. This new model is shown to exhibit the same features as previously reported experimental MEMS friction loop data. The correlation function of the surface roughness is shown to play a critical role in the modelling. It is experimentally obtained by probing the sidewall surfaces of a MEMS device flipped upright in on-chip hinges with an AFM (atomic force microscope). The addition of a modulation term to the model allows us to also simulate the effect of vibration-induced friction reduction (normal-force modulation), as a function of both vibration amplitude and frequency. The results obtained agree very well with measurement data reported previously.

## Introduction

With the invention of the friction force microscope (FFM) by Mate et al. [1], it has become possible to study the friction processes on the atomic scale that count as one of the fundamental aspects of everyday friction. The FFM (an atomic force microscope (AFM) that is sensitive to the lateral forces at the tip) can probe the interactions of an (almost) atomically sharp tip with individual atoms or a small part of a crystal lattice on the Ångstrom scale. It was found that regular, repeatable stick-slip behaviour of a contacting highest point (asperity) over the lattice of the other surface forms the very basis of the frictional processes as previously described [2–3]. To physically describe the stick-slip behaviour observed, the theories of Prandtl [4] and Tomlinson [5] were used [6–7]. This Prandtl–Tomlinson model has proven to be remarkably effective in describing atomic-scale friction.

Further research on atomic-scale friction has resulted in a wealth of information on atomic-scale friction processes, culminating in the prediction and discovery of extremely interesting processes like superlubricity (vanishing friction when crystal lattices do not match) [8–9] and thermolubricity (vanishing friction due to temperature-assisted hopping) [10–11]. Using the Prandtl–Tomlinson model and kinetic rate theory, it has been possible to describe the observed behaviour in simple theoretical terms.

The difference in length scales between the macroscopic and the atomic-scale regime is extremely important. Atomic scale friction experiments on atomically flat, non-reactive surfaces often show very low friction coefficients (e.g., ~0.01 for a tungsten tip on graphite [1]), while macroscopically, usually friction coefficients above 0.1 are encountered. Hence it is not directly clear how the atomic-scale friction coefficients relate to their macroscopic counterparts. This transition regime is also of practical significance: MEMS (micro-electromechanical systems) devices have contact forces, surface roughness and numbers of contacting asperities that position them right in this ‘knowledge gap’. In addition, their commercial success is severely hampered by continuing friction and wear problems [12].

The question is now how to describe friction on the larger scale of actual MEMS devices, which pair micrometer features and nanometer-scale surface roughness with nano- to micro-Newton forces. This friction is characterized by irregular, but repeatable, stick-slip motion. Can it still be described by the Prandtl–Tomlinson model? Work on rough surface friction has centred around dynamic critical phenomena by Fisher [13–14], Chauve et al. [15], and very recently by Fajardo and Mazo [16]. Friction of rough surfaces was also extensively studied by Persson et al. [17–18] using a dedicated contact mechanics model.

This paper first reviews typical MEMS friction measurements with our fully MEMS-based tribometer, showing the irregular, but repeatable, stick-slip motion of MEMS surfaces in contact. Then we extend the common Prandtl–Tomlinson model with a stochastic component to describe the surface roughness of the sliding MEMS. This model very effectively describes the statistical properties of the motion of the MEMS tribometer slider observed in several measurements. We also show the effect of vibration-induced friction reduction, both in the new theory and experiments.

## Results and Discussion

### MEMS tribometer friction measurements

**Figure 1:**
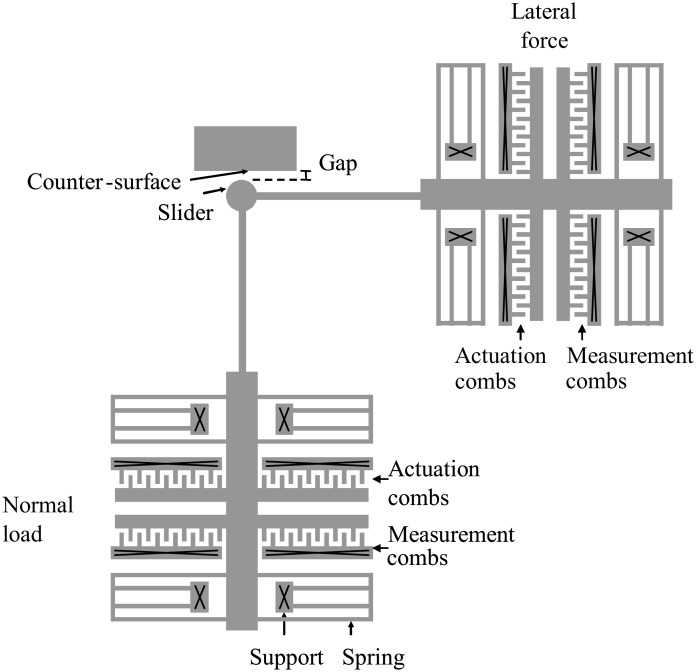
Schematic top view of the MEMS tribometer for studying microscale friction [19]. Several slider types have been investigated, such as the disc-shaped one in this figure. The experiments reported in the current paper have been performed with a square slider, resulting in two parallel sidewall surfaces sliding over one another. The slider surface is 20 μm by 2.0 μm, the counter-surface has the same 2.0 μm height but is much longer. Only the measurement shown in Figure 2 was performed with a small square slider of 4.0 μm by 2.0 μm. [Reprinted with permission from van Spengen, W. M.; Frenken, J. W. M. *Tribol. Lett.* **2007,** *28,* 149–156.]

**Figure 2:**
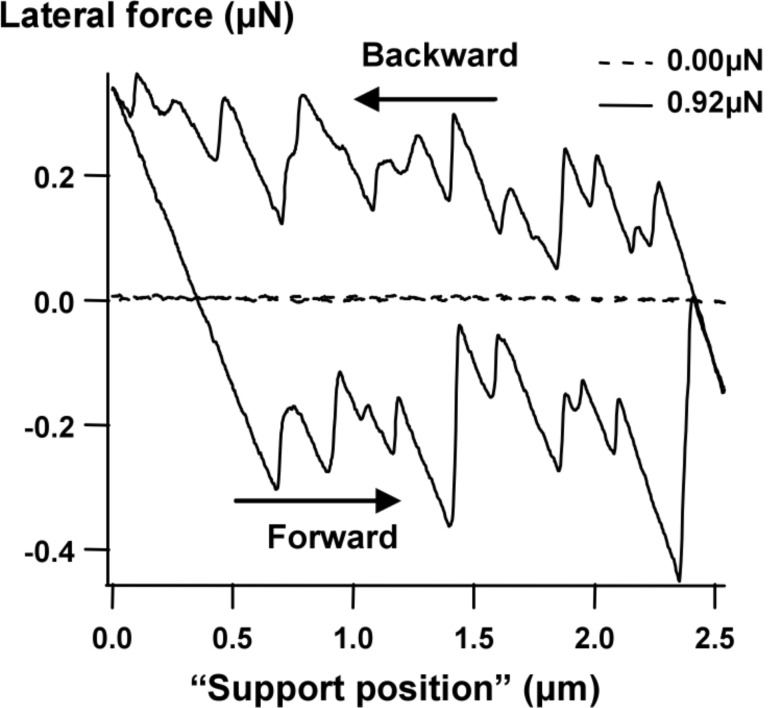
Typical 1000-cycle-average friction loops obtained with the tribometer of Figure 1 [19], at 27 °C and a relative humidity (RH) of 30%. The sliding speed was constant at 5 µm/s. Support position 0 μm is where the loop was started every cycle. This loop is an average over 1000 scans. The fact that the slips appear sharp means that there was no significant change to their position over these 1000 scans and hence no surface changes (which would indicate wear). [Reprinted with permission from van Spengen, W. M.; Frenken, J. W. M. *Tribol. Lett.* **2007,** *28,* 149–156.]

To investigate friction on the microscale, we have developed MEMS tribometer devices that can be used to perform friction experiments between their sidewalls [19]. They consist of two perpendicular ‘comb drive’ linear electrostatic actuators that can move a slider in two directions (Figure 1). One comb drive is used to press the slider against a counter-surface and to vary the normal load, and the other comb drive is used to slide the slider along the other surface. Although the device is mechanically comparable to the device described by Senft and Dugger [20], the readout mechanism is completely different. We use the capacitance change of a second set of comb fingers to detect the motion of the device [21]. This allows us to measure FFM-like dynamic friction loops showing the details of the interaction. A typical result with silicon MEMS sidewall surfaces in air, containing a native oxide, is shown in Figure 2. We observe irregular, but repeatable, stick-slip, on a length scale comparable to the lateral length scale of the surface roughness (to be quantified later).

To calibrate the forces measured, we need an accurate value for the spring constant of the device. This calibration has been implemented by designing two MEMS tribometers on the same chip, which have identical springs, but a known difference in mass. From the difference in resonance frequency we extract the spring constant, being 2.0 **±** 0.2 N/m for the device used in this study.

**Figure 3:**
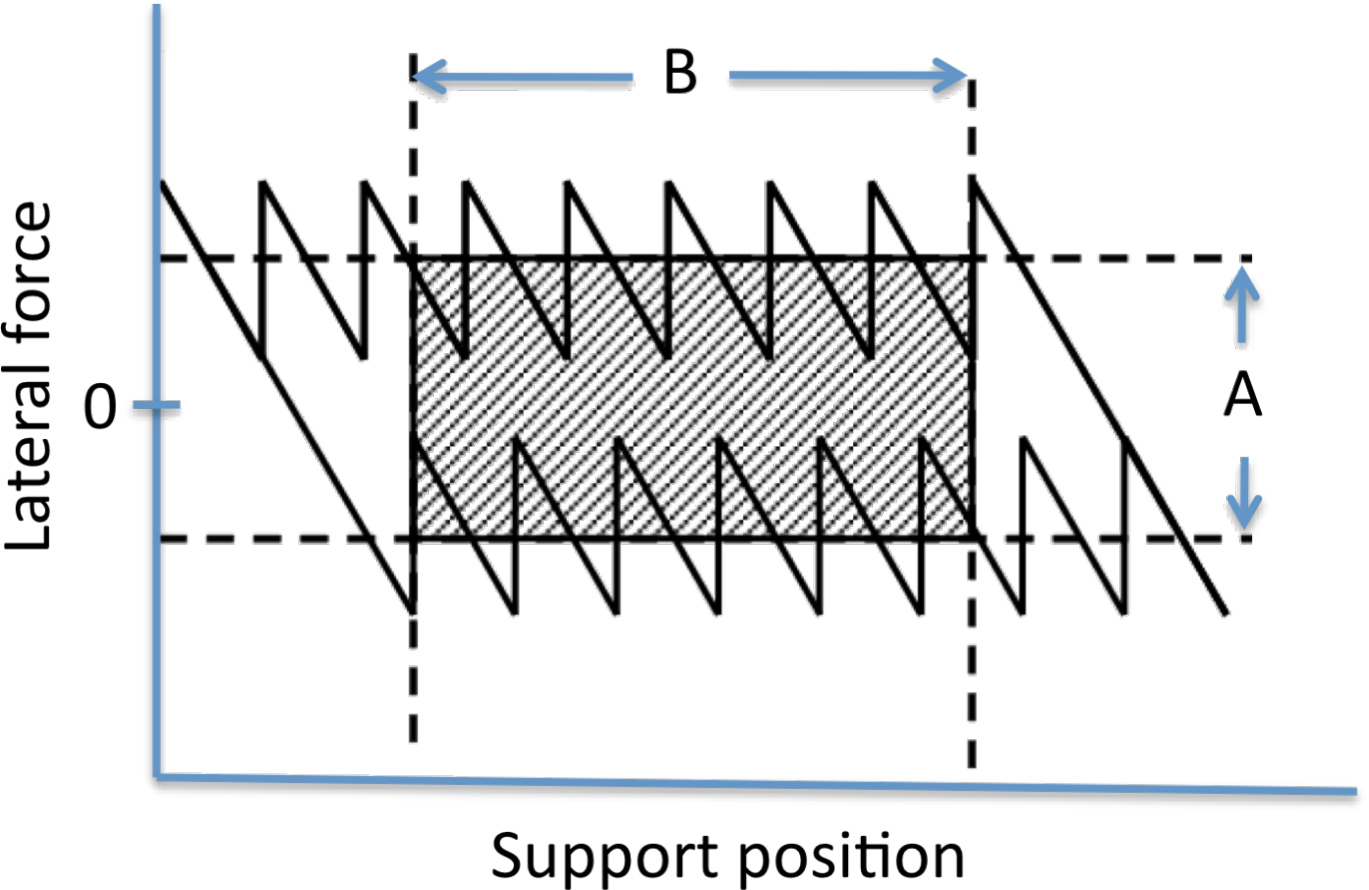
Determination of the average friction force. The area enclosed by the dashed lines provides the best estimate of the typical energy dissipated during sliding. The average friction force is obtained by dividing the energy contained in the shaded area by 2•B.

The area enclosed by the friction loop corresponds to the energy dissipated during the friction process. To obtain an accurate measure for the energy dissipation, we have cut off the side lobes of the friction loop, where the device becomes stuck in one direction, taking the average lateral force only when sliding in two directions takes place (Figure 3). From this dissipated energy, we calculated the average friction force such as plotted in the succeeding graphs, by dividing this energy by the distance slid.

**Figure 4:**
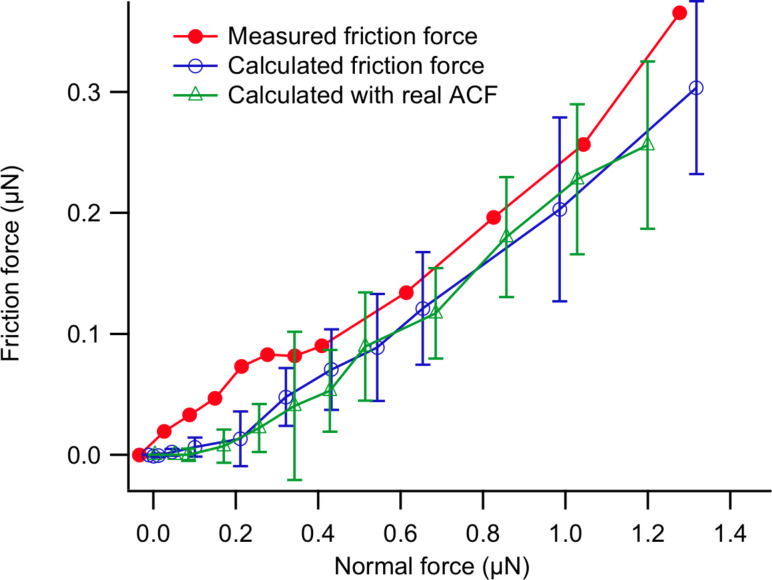
The average friction force (determined as depicted in Figure 3) as a function of the normal load is more or less linear on the scale of MEMS devices. The tests were conducted at 27 °C and a relative humidity of 30%. The fitted friction coefficient is 0.27. Indicated are also the calculated friction force based on an exponential autocorrelation function, with blue open circles, and with the measured autocorrelation function (‘real ACF’), indicated with green open triangles. The effect of the choice of autocorrelation function is very small.

In the measurements used for this paper, we systematically varied the normal force, while keeping the support position speed and environmental conditions constant. This resulted in a friction force that is more or less linear in the normal force, with a friction coefficient of 0.27 at a temperature of 27 °C and 25% RH (Figure 4). The fact that the friction force becomes zero at a negative apparent normal force is due to the contribution to the effective normal load of adhesion between the two surfaces.

### The new stochastic Prandtl–Tomlinson model

To describe the microscale irregular stick-slip behaviour, we have extended the well-known Prandtl–Tomlinson model [4–5,7], which is used to describe friction on the atomic scale, to include a microscale stochastic variation in the potential energy landscape. Normally, a periodic function is used, to describe the energy landscape with an atomic corrugation. In our case, the corrugations are much higher and dictated by the surface roughness. The characteristic length scale is related to the surface roughness correlation length of the MEMS sidewalls. We refer to our description as the ‘stochastic Prandtl–Tomlinson model’ (Table 1).

**Table 1:** Comparison of the atomic-scale and stochastic Prandtl–Tomlinson models.

	atomic Prandtl–Tomlinson model	stochastic Prandtl–Tomlinson model

**spring**	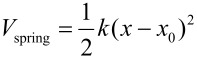	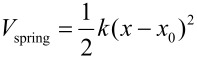
**surface corrugation**	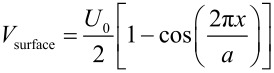	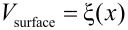

ξ(*x*) is a realization of a stochastic function, where *x* is the space variable (position). It obeys a Gaussian distribution function linearly related to the height of the surface and an exponential autocorrelation function with correlation length λ. This ‘recipe’ forms the simplest description of a stochastic process. To obtain the correlation length we need a model for the variations in interaction potential that the system of two surfaces will encounter when the surfaces slide with respect to one another.

If the MEMS tribometer would be a system in which the mean distance between the surfaces during sliding would be held constant, the contact area would fully change with the surface roughness. If the normal force would be held perfectly constant, the contact area would be constant instead (assuming a constant ‘bearing area’ [22]) and there would be no changes in the friction except the small changes expected on the atomic scale. But at the start of a slip event, the system is out of equilibrium and hence it is expected to behave intermediately between the two extremes mentioned. The natural length and amplitude scale of ξ(*x*) on which to expect changes are hence related to the length and amplitude scales of the surface roughness, even though the friction force is not determined by the work done against the normal force during sliding; the friction is much too high for this to be the dominating effect. In addition to the surface roughness, the elastic and inertial properties of the sliding surfaces and the whole system also contribute to the behaviour. This mode of friction is known in the literature as the ‘surface topology model of stick-slip’ [23].

**Figure 5:**
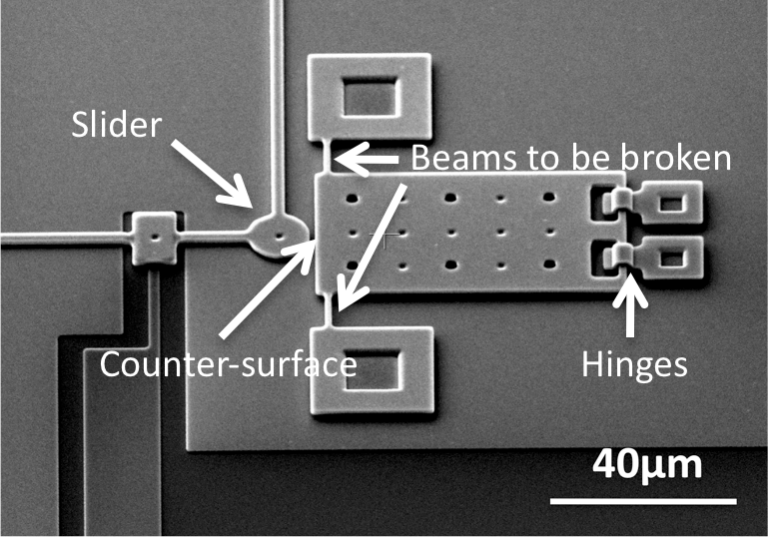
The counter-surface is held by two small beams. After the experiments, the beams can be broken and the counter-surface flipped upright in its hinges with a probe needle, allowing easy access with an AFM cantilever tip. The AFM has been used to measure the surface roughness (Figure 6) on the sidewall at the position where the arrow indicating ‘Counter-surface’ is pointing.

Based on this notion that ξ(*x*) is proportional to the surface roughness in MEMS, a measurement of the typical topology of the sidewall surface is required. We have made a special MEMS tribometer to do this, in which the counter-surface is supported with small beams and hinges instead of being directly fixed to the substrate. When the small beams are broken off with a probe needle, the counter-surface can be flipped upright and glued in place, so that conventional AFM can be used to quantitatively assess the sidewall roughness (Figure 5).

**Figure 6:**
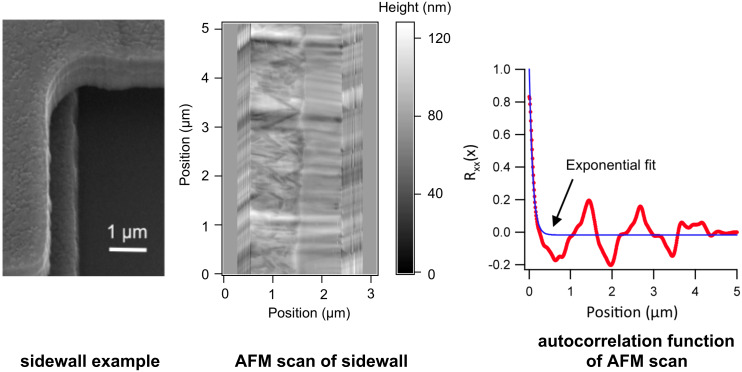
Autocorrelation function *R*_xx_(*x*) of a pristine sidewall surface measured with AFM, and theoretical exponential fit with a correlation length of 83 nm. The standard deviation on the height is 10.3 nm and the distribution is almost Gaussian. The long-range order is caused by the larger scale ripples. The result is that only one ripple may stick out significantly more than the others and hence friction is more localised than on a surface with a purely exponentially decreasing autocorrelation function. The sidewall measured with AFM is similar but not identical to the one used for the friction measurements.

The AFM data show several striking features: first of all, the polycrystalline silicon MEMS sidewall surfaces coming from the MEMSCAP MUMPS process are not perfectly random (Figure 6). Instead, two areas with apparently different roughness are visible, as is some long-range waviness on the micronscale. This surface structure is formed by the 2-step RIE (Reactive Ion Etching) process used for etching the structures from an initially continuous polycrystalline silicon film. These surface features are consistently there, from die to die, and from run to run, although they are, of course, also prone to statistical variation. As most probably, different parts of the surface will take part in the contact at the same time, and we require a 1-dimensional function; the autocorrelation function is obtained by adding all AFM scan lines taken in the direction of motion together, to obtain the graph on the right of Figure 6. This graph consists of the two different surface textures and the wiggly line separating the two. The result is an autocorrelation function with a fast, exponential decrease, and then some lower amplitude rippling that is not fully periodic but extends over a longer distance. The length scale of this ripple is most probably related to the grain size of the polycrystalline silicon that has been etched with RIE.

Using the sidewall AFM data, we have obtained a correlation length of 83 nm in the sliding direction for one individual surface (Figure 6). At very short distances at a correlation of 0.8 and higher, the measured value deviates from the exponential curve, showing that there is a lot of variation in the interaction energy at the nanoscale as well. To define the correlation length of the interaction potential realizations ξ(*x*), we need to take into account that there are two surfaces that both have this correlation length of 83 nm, and that the speed of change encountered when they slide over one another is then faster, and given by the square of the (normalized) individual autocorrelation functions. The correlation length of ξ(*x*) hence is λ = 41 nm, half the correlation length of the individual surfaces.

With the exponential autocorrelation function of Figure 6, and assuming a Gaussian distribution, we can now generate multiple mathematical 1-dimensional randomly rough surfaces as realizations of the so defined stochastic function ξ(*x*). As the correlation length is related to the surface roughness, the shape of the realization will not change with the normal load, as is also the case for the periodic Prandtl–Tomlinson model. The amplitude of ξ(*x*) is scaled linearly with the load with the scaling factor as the single fit parameter of the model.

### Friction loop simulations

The stochastic Prandtl–Tomlinson model was incorporated in an Igor Pro [24] software simulation of sliding rough surfaces with the statistical properties taken from the measurements described above. In this simulation, first the ‘surface roughness functions’, typically 50, are generated using ξ(*x*) with a scale in energy as the single fit parameter, namely the amplitude of ξ(*x*). For every surface, the following procedure is followed. First, the support position is set to 0, this is the first point on the left hand side. Combining the surface roughness function and the parabolic potential of the spring with support position 0, the momentary energy landscape is calculated. This also defines the lateral force scale on the vertical axis. Then a contact point is defined in the same place as the support position (zero at the left hand side). This is a single point, as the effect of having two surfaces has already been incorporated in ξ(*x*). This corresponds in a real measurement to the moment that the surfaces are brought together. Then the lowest energy point is determined, where the contact point can go monotonically (this is the essence of the Prandtl–Tomlinson model), and this point is given as the first position of the slider. From then on, every calculation cycle the support position is shifted by one point, the energy landscape is recalculated, and the lowest point in energy is evaluated where the contact point can go from its position in the previous cycle. This is repeated until the loop is completed. As a last step the trajectory of the contact point is evaluated for the first part of a second loop: from the last point in the cycle to the first time it encounters the original curve again. Indeed, the starting point of the second loop is not the same as that of the first, when the surfaces are brought into contact in which case the initial starting position for sliding is 0. By evaluating all realizations, one after the other, both a prediction can be made for the friction force that would be encountered in a typical experiment, and how much is would differ from one experiment to the other due to variations in the contacting surfaces.

**Figure 7:**
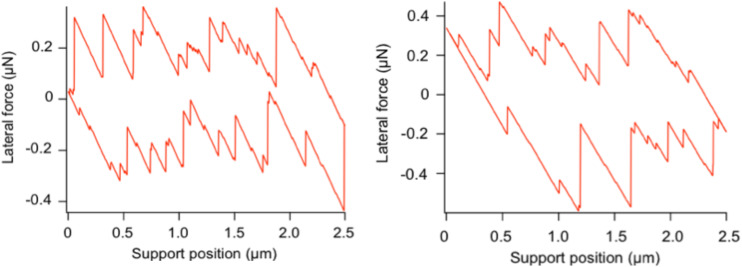
Examples of curves simulated with the stochastic Prandtl–Tomlinson model for two realizations of the same stochastic process, mimicking the experimental conditions of the measurement of Figure 2.

Simulated curves of the experiment of Figure 2 show a high degree of similarity to the measured data (Figure 7). The density of jumps, the typical jump length and the mean lateral force all agree well.

Friction loops for other normal loads were simulated as well. The lateral force for 50 loops and the standard deviation due to the stochastic nature of the realizations of the ‘surface roughness function’ ξ(*x*) are plotted in Figure 4 together with the measurements with blue open circles. The uncertainty bars in the calculation give the 1σ variation observed for different realizations of the surface profile. We see that the curve perfectly mirrors the behaviour of the experiment in Figure 4, however the whole curve is slightly offset to the right/down compared to the experiment and shows a regime of negligible friction at low normal loads, a region that we would associate in the traditional Prandtl–Tomlinson model with ‘superlubricity’. This is the case even though we have corrected for the 10 nN measured adhesion (adhesion measurements with the MEMS tribometer are detailed in [25]).

To investigate the effect of the long length scale ripples in the measured autocorrelation function on the outcome of the calculation shown in Figure 4, we have also performed the same simulation with the measured autocorrelation function instead of an ideally exponentially decaying one. These results were obtained for 25 friction loop simulations per normal force value and are shown with the green open triangles. There is no significant difference between the exponential and the ‘real’ autocorrelation simulations, and hence the effect of the ripples is negligible.

Because we have carried out MEMS measurements resembling force–distance curves (as described in [25]) as well as the friction measurements reported here, we are able to verify the zero-load point independent of the friction measurement. We can hence conclude that it is not allowed to shift the theoretical curve to the right to more closely fit the measurement data as one might be tempted to do, due to the assumption of the presence of a ‘superlubric’ regime. It seems that in hydrophilic silicon MEMS superlubricity does not take place. Instead a small extra friction force, most probably related to the water/hydrocarbons confined between and around the contacting asperities, has to be taken into account.

Just like the traditional periodic model, the stochastic Prandtl–Tomlinson model is phenomenological in the sense that it predicts the mechanical behaviour of the system, but does not say anything about the origin/amplitude of the corrugation, nor of the processes that cause the energy to really dissipate. In every slip, the stored elastic energy is suddenly released and contributes to a rise of the temperature of the sliding interface and eventually the whole MEMS device due to the thermalization of the phonons launched into the structure upon the impact of the contacting surface asperities [26].

The static shear strength itself is determined by OH-bridging forces between the surfaces, direct chemical Si–O–Si bonds between the surfaces (the rupturing of these bonds leads to wear of the surfaces in the long run), and/or possibly liquid water meniscus strain or even gluing by confinement induced solidified water [27].

### Vibration-induced lubricity simulations

**Figure 8:**
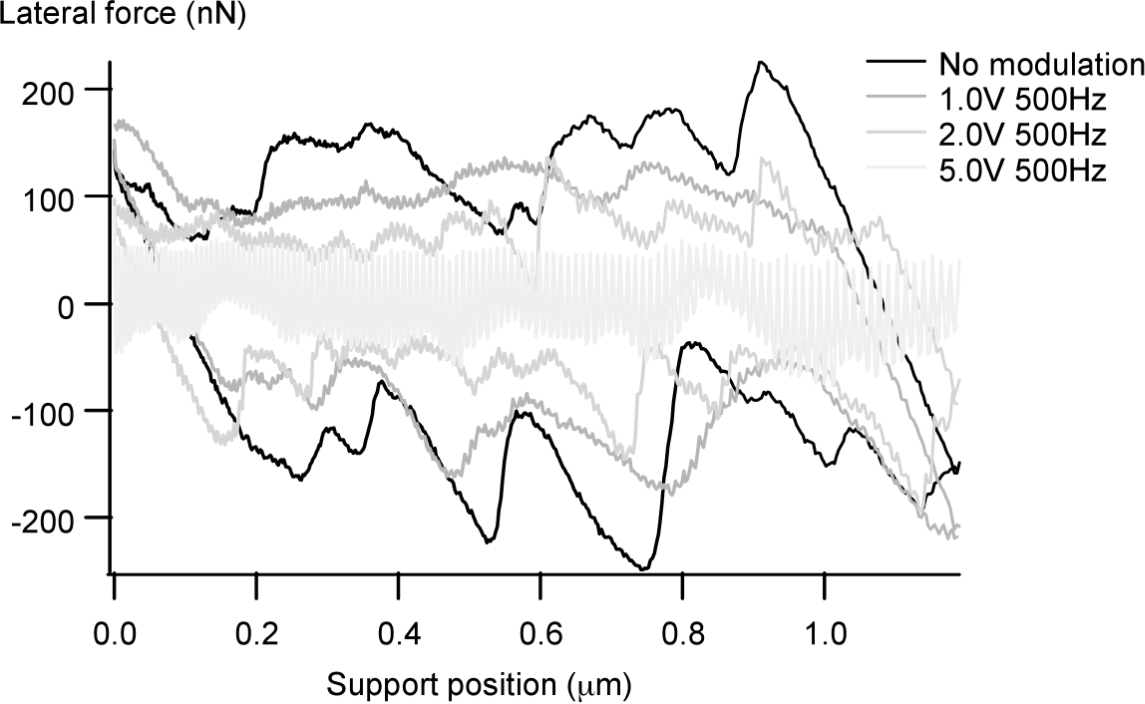
Modulation of the normal force at a frequency much higher than the frequency of the stick-slip events results in a significant decrease in the friction, and the appearance of a modulation signal in the lateral force. A voltage of 5.0 V is equivalent to 280 nN modulation peak–peak (linear scale) of the normal load. The average normal load is held constant at 50 nN. [Reprinted with permission from van Spengen, W. M.; Wijts, G. H. C. J.; Turq, V.; Frenken, J. W. M. *J. Adhes. Sci. Technol.* **2010,** *24,* 2669–2680.]

The energy barriers to be overcome in typical MEMS with sliding surfaces are much too large to take advantage of thermolubricity in order to lower friction. We have recently published the results of an experimental study in which we showed that, as in the case of thermal vibrations in thermolubricity, friction in MEMS can be significantly reduced by modulating the normal force, even when the average normal force is held constant. During the moments that the normal force is below the average, it is easier for the system to slip, and if it does, less energy is dissipated due to the smaller jumps involved. In [28], we presented the experimental results and a simple analytical model to predict the corresponding friction reduction. The friction measurement as a function of normal force modulation amplitude is replicated in Figure 8. The application of high-frequency vibrations to ease sliding has been reported on the macroscale already in 1959 [29], with the most recent investigation (in-plane motion) by Popov et al. [30]. Socoliuc et al. [31] have reported on atomic-scale experiments. In the latter case, frictionless sliding can even take place when the surfaces are still in slight contact.

With the new stochastic Tomlinson model presented here, it is now possible to fully simulate the effect of this modulation more precisely, both as a function of modulation amplitude and modulation frequency. The effect of modulation of the normal force can be simulated by multiplying the realization of the stochastic surface corrugation with this modulation. The way this is done is to first convert the modulation in time to a modulation in space during the sliding. The frequency of the modulation (e.g., 500 Hz) and the sliding speed (in these experiments and simulations sliding 1.2 μm back and forth in 0.5 s makes 4.8 μm/s) are combined. The spatial modulation period is then calculated as 4.8 μm·s^−1^/500 Hz = 9.6 nm. The momentary value of the corresponding sine wave is then multiplied with the energy landscape in agreement with the support position, so that one sine wave cycle is achieved for every 9.6 nm of support-position movement. The contact point can slide both forwards and backwards due to the modulation.

**Figure 9:**
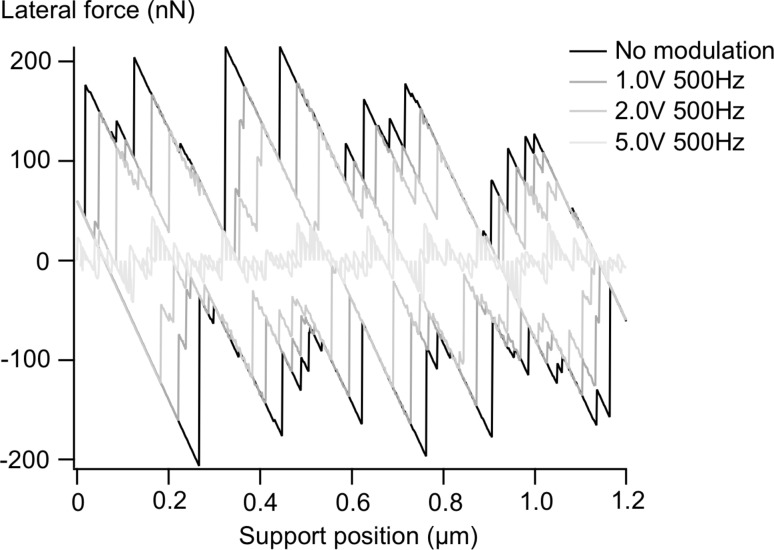
The major features of the experiment shown in Figure 8, including the amplitude reduction and the visibility of the modulation signal in the lateral force, are replicated in a simulation with the stochastic Prandtl–Tomlinson model of different modulation amplitudes. The peaks in the measurement appear blunter, most probably due to small-scale wear.

**Figure 10:**
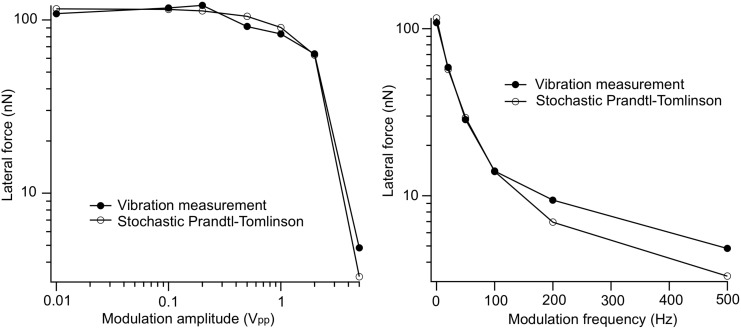
Calculated and measured friction reduction as a function of vibration amplitude (frequency held constant at 500 Hz, left figure) and frequency (5 V_pp_ amplitude, right figure).

The result is shown in Figure 9. The similarity between the simulation and the experiment is evident. The simulation replicates even the fact that a vibrational amplitude with the frequency of the modulation is visible in the lateral force at high modulation amplitudes (‘wobbling in the pits’), and that its envelope has a correlation with the surface roughness. Only in the simulation these effects are smaller than those experimentally observed, due to the fact that we are in this case close to the ‘superlubric’ regime in the model at low load. Figure 10 shows the expected trends of the friction reduction as a function vibration amplitude and frequency as calculated with the new model, as well as the measured curves; the agreement is excellent.

## Conclusion

The new stochastic Prandtl–Tomlinson model presented in this paper is a powerful tool to describe friction of nanometer-scale rough surfaces of MEMS. Although the model is fully phenomenological (it does not describe the physical processes that give rise to the energy dissipation) it is able to predict the important features of the typical motion observed of a polycrystalline silicon MEMS slider as it slides against an on-chip counter-surface of same material. This proves that the overall sliding behaviour is governed by the mechanical locking statistics due to the roughness of the surfaces. We have also shown that this new model can be easily extended with a term that describes the modulation of the normal force as present in vibration-induced friction reduction strategies. This extended model predicts the critical features of the vibration experiments very well.
